# Cluster randomized trial of reablement strategies targeting sarcopenia (ReStart-S) in long-term care settings

**DOI:** 10.1093/gerona/glag141

**Published:** 2026-05-29

**Authors:** Prabal Kumar, Shashikiran Umakanth, Kusumakshi Nayak, Olivier Bruyère, Vennila Jaganathan, Girish Nandakumar

**Affiliations:** Department of Physiotherapy, Kasturba Medical College Mangalore, Manipal Academy of Higher Education, Manipal, Karnataka, India; Department of Medicine, Dr TMA Pai Hospital, Udupi, Manipal Academy of Higher Education, Manipal, Karnataka, India; Department of Medical Laboratory Technology, Manipal College of Health Professions, Manipal Academy of Higher Education, Manipal, Karnataka, India; Research Unit in Public Health, Epidemiology and Health Economics, University of Liege, Liege, Belgium; Manipal College of Health Professions, Manipal Academy of Higher Education, Manipal, Karnataka, India; Department of Physiotherapy, Manipal College of Health Professions, Manipal Academy of Higher Education, Manipal, Karnataka, India; (Medical Sciences Section)

**Keywords:** Physical performance, Biomarkers, Exercise

## Abstract

**Background:**

Sarcopenia prevalence is high in long-term care settings (LTCS), yet existing guidelines often overlook this population. The Reablement Strategies targeting Sarcopenia (ReStart-S) program was developed to address this gap. This study evaluated its effects on muscle outcomes, physical performance, quality of life (QoL), and a blood biomarker.

**Methods:**

A cluster-randomized trial was conducted in LTCS across Udupi and neighboring districts. Sarcopenic older adults (≥ 60 years, Barthel Index ≥ 60, Mini-Cog ≥ 3, AWGS-2019 criteria) were recruited. LTCS were randomized into intervention (IG) and control (CG) groups. IG received a 6-week ReStart-S program, while CG continued usual activities. Outcomes at baseline, 6, 12, and 18 weeks included handgrip strength (HGS, primary outcome), skeletal muscle index (SMI), Short Physical Performance Battery (SPPB), SarQoL, and C-terminal Agrin Fragment (CAF; not assessed at 12 weeks). Linear mixed models evaluated group*time interactions with Bonferroni correction.

**Results:**

Of 12 LTCS screened, 7 were eligible; 78 participants enrolled (IG = 39; CG = 39). CG was older than IG (74.3 ± 9.4 vs 67.9 ± 6.0; *p* < .001). Significant group*time interaction was observed for HGS (*F* = 5.524; *p* = .001), improving at 12 (2.49; 95% CI, 1.16-3.82; *p* < .001) and 18 weeks (2.14; 0.79-3.48; *p* = .002). SPPB improved at 6, 12, and 18 weeks (all *p* < .001). SarQoL improved at all follow-ups (all *p* < .001). SMI improved at 18 weeks (0.20; *p* = .011). CAF decreased at 18 weeks (−61.77; *p* < .001).

**Conclusion:**

ReStart-S improved muscle strength, physical performance, and QoL, reduced CAF, and showed delayed muscle mass gains, supporting its role in sarcopenia care in LTCS.

**Clinical trial registration:**

The study was prospectively registered on October 20, 2022 on the Clinical Trial Registry-India (CTRI) platform. Trial registration number CTRI/2022/10/046680. The trial can be accessed at: https://ctri.nic.in/Clinicaltrials/showallp.php? mid1=71007&EncHid=&userName=CTRI/2022/10/046680

## Introduction

Sarcopenia, a common musculoskeletal geriatric medical condition, is characterized by a gradual reduction in muscle mass, strength, and overall physical performance.^1^^,^^2^ The prevalence is notably high among older adults residing in long-term care settings (LTCS), with reported rates ranging between 30% and 50%,^3^^,^^4^ reflecting the growing burden of this condition within the geriatric population. The health consequences of sarcopenia are diverse, encompassing an increased risk of falls, disability, reduced quality of life (QoL), poor oral health, loss of independence, and reduced physical performance.^5^ Among older adults in LTCS, sarcopenia is further linked to a two-fold higher risk of all-cause mortality.^6^ Therefore, it is crucial to address sarcopenia among older adults residing in LTCS through timely interventions and reablement strategies.^7^^,^^8^

National and international scientific groups have provided guidelines for the management of sarcopenia.^2^^,^^4^^,^^9–11^ The guidelines showed strong evidence of resistance training provided alone or as part of the multi-component exercise interventions to manage sarcopenia.^9–11^ However, when comparing older adults in LTCS to those living independently in the community, there are notable differences in their cognitive and physical abilities, QoL, polypharmacy and medical conditions they may have.^12^^,^^13^ Hence, the recommendation for the treatment of sarcopenia in LTCS should not be drawn only from the current practice guidelines, which are certainly more appropriate for community-dwelling older adults, necessitating a reablement program relevant to LTCS.

As per the internationally accepted definition, reablement is a goal-oriented, holistic approach to enhance an individual’s physical and functional abilities for independent daily living, regardless of diagnosis or settings.^14^ Evidence from previous research shows that multi-component exercise programs facilitated by physiotherapists are frequently implemented as part of a reablement approach for older adults residing in LTCS.^7^ For a longer impact of the reablement approach, the stakeholders (older adults, caregivers, administrators, physiotherapists, and medical professionals) should be consulted while designing a reablement program.^7^^,^^15^ Co-design is a collaborative approach that involves researchers, end-users, and stakeholders to tailor interventions to the specific needs of the target population.^16^ To optimize the implementation of evidence-based interventions according to the priorities and preferences of all stakeholders, enabling designed solutions to achieve maximum feasibility and sustainability, the co-design approach is the mainstay.^17^

In accordance with this requirement, we recently co-designed a program entitled Reablement Strategies targeting Sarcopenia (ReStart-S).^18^ This program is a comprehensive, multi-component exercise intervention specifically designed for sarcopenic older adults living in LTCS. The details of this program and the steps followed in co-designing have been documented in a prior publication.^18^ ReStart-S is structured as a progressive 6-week exercise regimen. Drawing on evidence from a comprehensive literature review and stakeholder engagement, the program includes four key components: resistance training, aerobic/endurance exercises, balance training (static and dynamic), and stretching. It adheres to the Frequency, Intensity, Time, and Type (FITT) principle, ensuring structured and individualized exercise delivery according to the sarcopenia status, including possible sarcopenia, sarcopenia, and severe sarcopenia.

Preliminary findings indicate that this program is feasible for implementation among sarcopenic older adults in LTCS.^18^ The LTCS in the Indian context differs from Western care models in terms of rehabilitation support, infrastructure, available resources, and care practices, with substantial variation across regions and communities.^19^^,^^20^ These contextual differences may influence adherence and reablement outcomes, highlighting the importance of evaluating the effects of ReStart-S on the targeted population. Thus, this study aims to look at the effect of the ReStart-S program on muscle strength, physical performance, muscle mass, blood-based biomarkers, and QoL among sarcopenic older adults residing in LTCS. We hypothesize that the ReStart-S program will improve the muscle parameters (strength and mass), physical performance, and QoL and decrease the biomarker level. The objective and hypothesis are targeted at the individual participant level; however, the randomization was done at the cluster level.

## Material and methods

### Trial design and settings

The present study is a cluster randomized trial with two arms, and its protocol has been published.^21^ The trial design has been chosen to prevent contamination of the data. The trial took place among residents of eligible long-term care facilities for older adults located in Udupi and its neighboring districts in Karnataka, India. The CONSORT 2010: extension to cluster randomized trial statement,^22^ has been followed for reporting to ensure clinical trial transparency (Supplementary Material 1). The Institutional Ethics Committee has granted approval for this study [IEC1: 100/2022]. The trial is registered [CTRI/2022/10/046680]. The study duration was from September 2023 to July 2024.

### Study participants and eligibility criteria

#### Selection criteria for clusters

Clusters were assessed for their eligibility based on the inclusion and exclusion criteria mentioned below:

*Inclusion criteria:* (1) LTCS for older adults, (2) Udupi and neighboring districts (Dakshina Kannada, Uttara Kannada, Chikkamagaluru, Shivamogga).

*Exclusion criteria:* The cluster providing (1) adult day care, (2) hospice care, and (3) settings that provide respite care were excluded.

#### Selection criteria for participants

Participants were assessed for their eligibility following the inclusion and exclusion criteria mentioned below:

*Inclusion criteria:* The participants must (1) be of either gender, (2) be aged 60 years or older, (3) have a Barthel index score of 60 points or higher, (4) Mini-Cog score ≥ 3, (5) meet the criteria for sarcopenia according to the Asian Working Group for Sarcopenia 2019 (AWGS-2019) guidelines (2), which include: (a) Skeletal muscle index (SMI): less than 7.0 kg/m^2^ for males and less than 5.7 kg/m^2^ for females, (b) Handgrip strength (HGS): less than 28.0 kg for males and less than 18.0 kg for females, and (c) Short Physical Performance Battery (SPPB): a score of 9 or lower.

*Exclusion criteria:* Participants were excluded for the following reasons: (1) being critically or terminally ill or having an active infection, (2) having a pacemaker or any metal implants, (3) being confined to bed or reliant on a wheelchair, (4) experiencing an acute onset (within the last thirty days) of neurological, renal, cardiorespiratory, or orthopedic conditions.

### Sample size

The study has been powered according to the primary outcome: HGS. To determine the sample size, we used a comparison of the mean formula (2 × (Z_1-α/2_ + Z_1-β_)^2^ × (σ)^2^/(µ_d_)^2^ and considered the standard deviation of grip strength (4.5 kg) and the minimum clinically important difference (3.90 kg) based on previous randomized controlled trials.^23^^,^^24^ With a significance level (Z_1-α/2_) of 5% and power (Z_1-β_) of 80%, we arrived at a sample size of approximately 21 participants per arm. However, after factoring in a design effect of 1.5, the estimated sample size increased to around 32 participants per arm. Accounting for a potential dropout rate of 20%, we arrived at a total of 39 participants in each arm as an adequate sample size for the study.

### Study procedure, randomization, and blinding

The clusters (LTCS) were identified using purposive sampling and assessed for eligibility. The permission letter (cluster-level consent) was obtained from the eligible LTCS management. All residents at the eligible LTCS underwent screening after a comprehensive explanation of the study procedures, and informed consent (participant-level consent) was obtained in writing. Participants within the clusters were recruited using convenience sampling, and eligible participants were stratified into (1) possible sarcopenia (reduced HGS or physical performance), (2) sarcopenia (loss of skeletal muscle mass (SMM) plus reduced HGS or reduced physical performance), and (3) severe sarcopenia (loss of SMM plus reduced muscle strength plus reduced physical performance). Baseline data (week 0) were collected from all eligible residents of the LTCS. Given that the clusters had widely varying numbers of residents (ranging from *n* = 5 to *n* = 20), the blocking approach became infeasible. In response, the clusters were pragmatically re-grouped to balance the intervention and control arm in terms of overall sample size. Subsequently, the LTCS were randomized into the intervention group (IG) and control group (CG) using a lottery-based probability randomization method. Each LTCS was assigned a unique identification number, and the allocation was determined by randomly drawing the numbers from an opaque container. The randomization procedure was performed independently by a researcher who was not involved in participant recruitment, baseline assessment, intervention delivery, or outcome assessment, thereby minimizing allocation bias.

**Figure 1:**
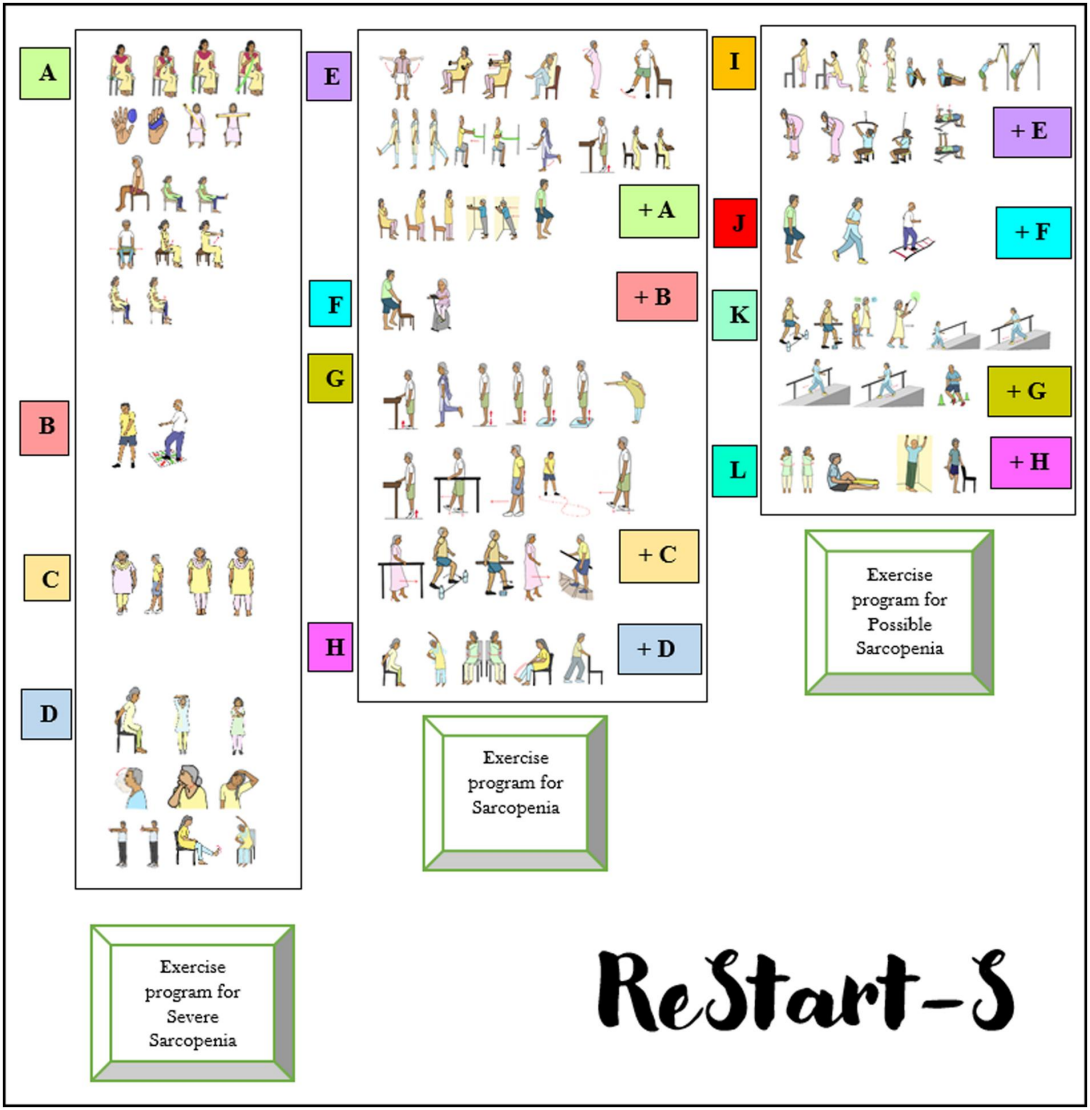
Reablement strategies targeting sarcopenia (ReStart-S) program.

The ReStart-S program was delivered to the participants in the IG. The ReStart-S program is a 6-week multi-component exercise program that tailors exercises based on sarcopenia status: severe sarcopenia, sarcopenia, and possible sarcopenia. For severe sarcopenia, exercises include A (resistance), B (aerobic), C (balance), and D (stretching). Sarcopenic participants perform these along with additional exercises: E (resistance), F (aerobic), G (balance), and H (stretching). Possible sarcopenia participants perform all previous levels plus: I (resistance), J (aerobic), K (balance), and L (stretching), resulting in a progressive increase in exercise volume, as illustrated in Figure 1. The details of the program have been published elsewhere.^18^ The eligible participants were made to sit in the group as per the sarcopenia status: possible sarcopenia, sarcopenia, and severe sarcopenia. The ReStart-S program has been delivered as per the FITT principle. The frequency was twice per week for a total duration of six weeks. The exercise intensity was assessed and progressed using the 6-20 rating of perceived exertion scale (RPE). The duration of the exercise session ranges from 60 minutes to 90 minutes, of which most of the time was assigned to resistance exercises. The progression of the exercise was done week-wise, in the form of increasing the RPE/volume of the exercise/number of exercises. All the exercises were performed under the supervision of the physiotherapist. The various exercise equipment, like dumbbells (1, 2, and 3 kg), weight disks (2 and 3 kg), resistance bands and tubes (of multiple colors), hand grip exerciser kit (grip exercises, ring squeezer, and ball squeezer), were used to deliver the resistance exercises. The cones were used to mark the points on the walkway to do the aerobic and endurance exercises. The figure of eight, obstacle clearances and ladders were used for balance training along with other exercises. The stretching exercises were included as the warm-up and cool-down sessions. Participants were motivated while exercising using verbal motivation vocabulary, such as doing good, great, go on, etc. The participants were also involved in fun-based activities (like ball throwing/catching, ball kicking, throwing a ball to the target, and hitting the ball to the target using the wand) to ensure retention for the intervention period. The complete reporting of the intervention has been done using a Consensus on Exercise Reporting Template (CERT) published elsewhere. The participants in the CG were asked to continue with their daily usual activities, which included self-care tasks, leisure and social interactions, and routine mobility within the facility, without participation in any structured or supervised exercise program during the study period. After completion of the 6-week supervised ReStart-S program, participants in the IG were encouraged to continue the same exercise routine independently until the final follow-up at week 18. No supervised sessions were provided beyond week 6, and adherence during this period was based on self-report, without formal monitoring.

Blinding the allocation of groups from the therapist responsible for the intervention was not feasible, as the PhD scholar delivering the protocol was directly involved in its administration. Similarly, blinding the intervention from the participants was not possible, as they were actively engaged in receiving it.

### Outcome measure and assessment

The outcome pertains to the individual participants’ level. The primary outcome measure was HGS, and secondary outcome measures included physical performance, SMI, C-terminal Agrin Fragment (CAF) biomarker level, and QoL. Primary and secondary outcomes were measured at individual participant levels on four evaluation time points at week 0 (baseline), first follow-up at week 6 (post-intervention), second follow-up at week 12 (except CAF), and third follow-up at week 18, shown in Table 1. The primary endpoint for evaluating the effect of the 6-week ReStart-S program was set at week 6 (post-intervention), as the intervention lasted for 6 weeks. However, follow-up assessments were conducted at weeks 12 and 18 to examine the sustainability and delayed effects of the intervention on sarcopenia-related parameters.

**Table 1:** Timeline for the measurement of primary and secondary outcomes of the study participants.

	Measurement	Week 0 (baseline)	First follow-up at week 6 (post intervention) period	Second follow-up at week 12	Third follow-up at week 18
**Primary outcome**	Hand grip strength (kg)	×	×	×	×
**Secondary outcomes**	Short Physical Performance Battery (score)	×	×	×	×
	Skeletal muscle index (kg/m^2^)	×	×	×	×
	Sarcopenia quality of life questionnaire (score)	×	×	×	×
	C-terminal agrin fragment (pg/mL)	×	×	–	×

The HGS was measured using a JAMAR Plus Digital Hand Dynamometer (Patterson Medical Ltd., Cedarburg, United States of America). Participants were asked to identify their dominant hand, which was defined as the hand preferred for daily activities such as writing or eating. Before data collection, each participant was given a trial run using their non-dominant hand to familiarize themselves with the procedure. HGS was then measured on the dominant hand following the standard procedure.^25^ Two trials were performed, and the best of the trial was recorded. HGS is a well-established predictor of disability, morbidity, and mortality, and is one of the key components of the AWGS 2019 guidelines for diagnosing sarcopenia. HGS assessment is a reliable and valid procedure among healthy participants as well as across various clinical populations.^26^ We hypothesize that the intervention would lead to an increase in the HGS.

Physical performance was assessed using the SPPB, a standardized test that evaluates balance (in three different positions: feet together, semi-tandem, and tandem), timed walking (over a 4-meter course), and lower extremity strength (performing five sit-to-stand repetitions as quickly as possible) in older adults. The test score ranges from zero to twelve; each component’s maximum score is four.^27^ A total score of less than or equal to 9 indicates sarcopenia as per the AWGS 2019 criteria (2). Research suggests the test has test-retest reliability Intra-class correlation coefficient (ICC) 0.87 to 0.93, with an improvement of 0.54 to 1.42 points is clinically significant.^28^

SMI was estimated using Bioelectrical Impedance Analyzer (BIA)-derived predictive equations. Omron Karada Scan HBF-375 BIA (Omron Healthcare Co., Ltd, Kyoto, Japan) has been used in the study that provides skeletal muscle ratio (%), which reflects the proportion of SMM relative to body weight. Using this parameter, SMM (kg) was derived as: SMM (kg) = [skeletal muscle ratio (%) × body weight]/100. Further, the SMI (kg/m^2^) was derived as: SMI (kg/m^2^): SMM (kg)/height^2^. Participants were instructed to void their bladder, refrain from consuming food or drink for at least two hours prior to the measurement and avoid physical exercise for eight hours. The BIA platform was cleaned with an alcohol rub before each use. Required inputs, including age, gender, and height, were entered into the device. Participants were then instructed to stand on the designated foot marks and hold the hand device at 90-degree shoulder flexion for 5-10 s.^29^ After the procedure, participants stepped off the device, and the obtained readings were recorded.

A blood-based biomarker, the CAF, pg/mL was measured in the study. Previous scoping reviews have indicated that higher levels of CAF are associated with sarcopenia.^30^ The procedure was performed by an expert from the Department of Medical Laboratory Technology. Participants were asked to fast overnight, and blood samples were collected in the morning from the cubital vein using vacutainers containing the anticoagulant ethylenediamine tetraacetic acid (EDTA). The vacutainers were stored under controlled temperature conditions and transported to the laboratory. Blood samples were transported in a specimen box containing cold packs to maintain cold temperature (2-8 °C) and centrifuged at 1000 *g* (3000 rpm) for 10 min at 4 °C using a refrigerated centrifuge (Thermo Fisher Scientific-Sorvall™ ST 8, Osterode am Harz, Germany). Following this, 100 µL alliquotes of plasma were prepared in cryovials and stored at −20 °C for 15 days-1 month, until analysis. The analysis was performed as per the procedure prescribed by the ELISA kit developers. The Human CAF ELISA Kit (Cat: ELK9769) by ELK Biotechnology was used, and the Sandwich enzyme immunoassay test principle was applied. The Multiskan FC (Version 1.01.16) and SkanIt Software for Microplate Readers RE, ver. 5.0.0.42 was used to read the plate, and readings were noted.

Sarcopenia is associated with reduced QoL,^31^ and participants QoL was assessed using the self-administered Sarcopenia Quality of Life Questionnaire—Kannada version (SarQoL-Kannada).^32^ The questionnaire consists of 55 items, consolidated into 22 questions, rated on a 4-point Likert scale. These items are categorized into seven domains: Physical and mental health, Locomotion, Body composition, Functionality, Activities of daily living (ADL), Leisure activities, and Fears. The total score ranges from zero to one hundred, with higher scores indicating better QoL. Participants were provided with a printed copy of the questionnaire to record their responses. The responses were then encoded into an online account on the SarQoL website for analysis. The SarQoL-Kannada has demonstrated high internal consistency (Cronbach’s alpha = 0.904) and excellent test-retest reliability (intraclass correlation coefficient = 0.97, 95% CI, 0.92-0.98).^32^ It was hypothesized that the intervention would lead to improvements in the SPPB score, SMI, QoL, and a reduction in CAF concentration.

### Data analysis

All statistical analyses were conducted using JAMOVI version 2.4.14. Descriptive statistics were used to summarize the demographic and baseline characteristics of the participants in both groups. Continuous variables were expressed as means with standard deviations. Categorical variables were expressed as frequencies and percentages. Independent *t*-tests (for continuous data) and chi-square tests (for categorical data) were performed to assess differences in baseline characteristics between the IG and CG. Given the repeated measurements of outcomes across multiple time points (0, 6, 12, and 18 weeks), a linear mixed-effects model (LMM) was used to account for both within- and between-subject variability. The models included fixed effects for the group (IG vs CG), time (weeks 0, 6, 12, and 18), and the group × time interaction. Individual and cluster intercepts were included as random effects represented by the ICC. To explore the effect of the covariate variables (age, physical activity, months of stay in LTCS, and family visits per year), which were significantly different at the baseline, an adjusted LMM was also performed. Effect sizes were estimated using partial eta-squared (η^2^*p*) to evaluate the magnitude of intervention effects, with values of 0.01, 0.06, and 0.14 representing small, medium, and large effects, respectively. Given that the intervention was stratified based on sarcopenia status, interaction analyses using repeated measures ANOVA were specifically performed to evaluate potential heterogeneity in treatment effects. The Time × Group × Sarcopenia status interaction term was included to determine whether intervention effects varied across sarcopenia categories, thereby addressing potential confounding due to differential intervention exposure. Post-hoc pairwise comparisons with Bonferroni correction were conducted to explore specific differences between time points within and between groups at particular time points. A *p*-value of less than or equal to .05 was considered statistically significant.

## Results

**Figure 2:**
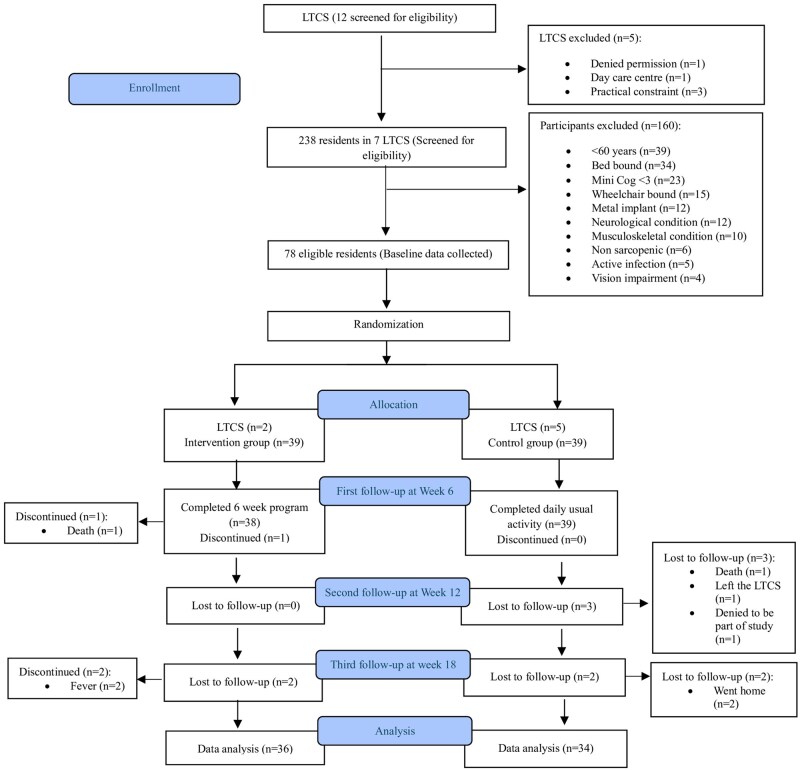
Participants flowchart of the study [CONSORT 2010].

A total of twelve (*n* = 12) LTCS were screened for eligibility. Five (*n* = 5) settings were excluded, one (*n* = 1) denied permission, one (*n* = 1) was a daycare center, and three (*n* = 3) were excluded due to practical constraints. A total of two hundred thirty-eight (*n* = 238) residents were screened in the seven eligible LTCS. A total of seventy-eight (*n* = 78) sarcopenic older adults were found eligible. The participants were allocated into an IG (2 LTCS, *n* = 39) and a CG (5 LTCS, *n* = 39). Among them, seventy-seven (*n* = 77) completed the first follow-up, seventy-four (*n* = 74) completed the second follow-up, and seventy (*n* = 70) completed the third follow-up. The participant flow is presented in the CONSORT flow diagram Figure 2.

### Demographic characteristics of the participants

The mean age of the participants was significantly (*p* < .001) different between the groups, with higher in the control group (74.30 ± 9.39) than in the intervention group (67.87 ± 6.01). The baseline grip strength was found to be higher in the control group (15.6 ± 6.42) than in the intervention group (13.4 ± 6.92); however, it was not significantly different (*p* = .130). The sarcopenic status (severe sarcopenic, sarcopenic, and possible sarcopenic) of the participants between the control and intervention groups did not differ significantly (*p* = .510). Regarding marital status, the control group had a higher proportion of married participants (25.6%) and widowed individuals (43.6%), whereas the intervention group had a higher proportion of unmarried individuals (46.2%). Education levels varied significantly between the groups (*p* < .001), with the CG having a higher proportion of participants with secondary or higher education, while the IG had more participants with only primary education. The details of the demographic characteristics can be found in Table 2.

**Table 2:** Demographic characteristics of the participants (*N* = 78).

Variable	CG (*n* = 39)	IG (*n* = 39)	*p*-Value
**Age (mean ± *SD*; years)**	74.30 ± 9.39	67.87 ± 6.01	<.001^a^
Male	71.4 ± 8.51	69.4 ± 7.03	
Female	77.0 ± 9.55	66.8 ± 5.09	
**Gender [*n* (%)]**			.495
Male	19 (48.7)	16 (41.1)	
Female	20 (51.3)	23 (58.9)	
**Weight (mean ± *SD*; kg)**	55.70 ± 10.1	51.6 ± 11.9	.108
Male	59.1 ± 7.49	57.0 ± 9.76	
Female	52.5 ± 11.3	47.9 ± 12.1	
**Height (mean ± *SD*; cm)**	159 ± 10.1	154 ± 8.64	.01^a^
Male	168 ± 5.58	161 ± 6.77	
Female	151 ± 5.11	149 ± 6.34	
**BMI (mean ± *SD*; kg/m^2^)**	22.0 ± 3.77	21.7 ± 4.14	.730
Male	21.1 ± 31.9	22.0 ± 3.11	
Female	22.9 ± 4.15	21.6 ± 4.79	
**Hand dominance [*n* (%)]**			.152
Right	37 (94.9)	39 (100)	
Left	2 (5.1)	0	
**Family visits (mean ± *SD*; visits/year)**	2.05 ± 2.54	0.0 ± 0.00	<.001^a^
Male	2.21 ± 2.78	0.00 ± 0.00	
Female	1.90 ± 2.36	0.00 ± 0.00	
**LTCS months of stay (mean ± *SD*; months)**	54.40 ± 69.70	20.40 ± 18.20	.004^a^
Male	34.9 ± 39.8	14.4 ± 8.21	
Female	73 ± 86.4	24.6 ± 22.0	
**PA (mean ± *SD*; min/d in a year)**	8.33 ± 12.9	2.82 ± 4.70	.015^a^
Male	12.1 ± 15.8	0.93 ± 2.72	
Female	4.75 ± 8.35	4.13 ± 5.36	
**Marital status [*n* (%)]**			.322
Married	10 (25.6)	6 (15.4)	
Unmarried	12 (30.8)	18 (46.2)	
Widowed	17 (43.6)	14 (35.9)	
Divorced	0	1 (2.6)	
**Education level [*n* (%)]**			<.001^a^
Illiterate	0	12 (30.8)	
Primary	15 (38.5)	22 (56.4)	
Secondary	14 (35.9)	4 (10.3)	
Higher	10 (25.6)	1 (2.6)	
**ADL (Independent/dependent; *n*)**			
Bathing	38/1	25/14	<.001^a^
Dressing	38/1	30/9	.007^a^
Toileting	39/0	38/1	.314
Transferring	39/0	38/1	.314
Continence	39/0	39/0	NaN^b^
Feeding	39/0	39/0	NaN^b^
**History in the last one year (yes/no; *n*)**			
Smoking	2/37	1/38	.556
Alcohol	1/38	2/37	.556
Falls	8/31	0/39	.003^a^
Fracture	1/38	1/38	1
Hospitalization	7/32	4/35	.329
**Comorbidities [*n* (%)]**			.164
Diabetes mellitus	5 (6.4)	4 (5.1)	
Hypertension	10 (12.8)	17 (21.8)	
Diabetes and hypertension	8 (10.3)	4 (5.1)	
Gastric disorders	3 (3.8)	2 (2.6)	
Epilepsy	1 (1.3)	1 (1.3)	
Asthma	0 (0.0)	2 (2.6)	
Dermatological disorders	0 (0.0)	3 (3.8)	
None	12 (15.4)	6 (7.7)	
**Medications [*n* (%)]**			.164
Antidiabetics	5 (6.4)	4 (5.1)	
Antihypertensives	10 (12.8)	17 (21.8)	
Antidiabetics and antihypertensives	8 (10.3)	4 (5.1)	
Antacids	3 (3.8)	2 (2.6)	
Antiepileptic drugs	1 (1.3)	1 (1.3)	
Bronchodilators	0 (0.0)	2 (2.6)	
Topical corticosteroids	0 (0.0)	3 (3.8)	
None	12 (15.4)	6 (7.7)	
**SMI (mean ± *SD*; kg/m^2^)**	5.14 ± 1.01	5.09 ± 1.15	.848
Male	5.79 ± 0.71	5.78 ± 0.96	
Female	4.52 ± 0.85	4.61 ± 1.04	
**HGS (mean ± *SD*; kg)**	15.6 ± 6.42	13.4 ± 6.92	.144
Male	18.7 ± 7.06	18.9 ± 6.35	
Female	12.7 ± 4.07	9.54 ± 4.22	
**SPPB (mean ± *SD*; score)**	7.08 ± 2.77	8.33 ± 2.72	.047^a^
Male	7.37 ± 2.95	8.69 ± 2.89	
Female	6.80 ± 2.63	8.09 ± 2.63	
**Sarcopenia status [*n* (%)]**			.510
Possible sarcopenia	4 (10.3)	6 (15.4)	
Sarcopenia	10 (25.6)	13 (33.3)	
Severe sarcopenia	25 (64.1)	20 (51.3)	
**SarQoL (mean ± *SD*; score)**	49.7 ± 13.5	46.3 ± 14.3	.292
Male	52.6 ± 16.2	47.0 ± 12.6	
Female	46.9 ± 10.1	45.8 ± 15.6	
**CAF (mean ± *SD*; pg/mL)**	77.0 ± 33.3	79.2 ± 13.9	.704
Male	74.3 ± 27.1	75.8 ± 13.4	
Female	79.5 ± 38.8	81.5 ± 14.1	

Abbreviations: LTCS, Long-term care settings; PA, Physical activity; BMI, Body mass index; SMI, Skeletal muscle index; HGS, Hand grip strength; SPPB, Short Physical Performance Battery; SarQoL, Sarcopenia Quality of Life questionnaire; CAF, C-terminal agrin fragment; ADL, activities of daily living; CG, control group; IG, intervention group. Independent *t* test for the continuous variables (age, LTCS, PA, number of family visits, weight, height, BMI, SMI, HGS, SPPB, SarQoL, CAF).

Chi-square test for categorical variables (gender, sarcopenia status, marital status, education level, ADL, history in last one year of smoking, alcohol, fracture, fall, hospitalization, hand dominance).

a Significant difference between control group (CG) and intervention group (IG).

b All observations are tied up.

#### HGS (Primary outcome)

Baseline mean HGS (IG = 13.4 ± 6.92; CG = 15.6 ± 6.42) were comparable between both groups (*p* = .144). There found to be a significant group*time interaction (*F* = 5.524, *p* = .001, η^2^_p_ = .073), indicating that the change in HGS over time was different between IG and GC, with a moderate effect size. At 12 weeks, the IG showed a significantly greater increase in HGS compared to the CG (estimate = 2.49; 95% CI, 1.16 to 3.82, *p* < .001), and this effect persisted at 18 weeks (estimate = 2.14; 95% CI, 0.79 to 3.48), *p* = .002). However, the difference between groups was not significant at 6 weeks (estimate = 1.02; 95% CI, −0.27 to 2.31, *p* = .123). Individual variance was 33.60 (*SD* = 5.80, ICC = 0.889), mostly within clusters. Cluster variance was 9.34 (*SD* = 3.06, ICC = 0.691). Residual variance (4.19, *SD* = 2.05) suggests the model explained most HGS variability. Upon adjusting for covariates, the effect estimates for group-by-time interactions remained largely unchanged, indicating robustness of the intervention effects. Specifically, the intervention group continued to show significantly greater improvements in HGS at 12 weeks (estimate = 2.495, *p* < .001) and 18 weeks (estimate = 2.140, *p* = .002) compared to the control group. The post hoc comparison details have been provided in detail in Table 3. The interaction analysis using repeated measures ANOVA demonstrated that the three-way interaction (Time × Group × Sarcopenia status) was not statistically significant (*p* = .389), suggesting that the effect of the intervention over time did not differ across sarcopenia categories. These findings indicate that, despite stratification of the intervention, its effectiveness was consistent across different sarcopenia statuses, thereby reducing concerns regarding treatment heterogeneity influencing the outcomes.

**Table 3:** Changes in outcomes following reablement strategies targeting sarcopenia (ReStart-S) program.

Outcomes	Within-group			Between-group	
	IG (*n* = 39)	CG (*n* = 39)	*p*_bonferroni_ (IG/CG)	Estimate (95% CI)	*p*-Value
	Difference (*SE*)	Difference (*SE*)			
**HGS**					
Week 0					
Week 6	1.199 (.469)	0.179 (0.463)	.292/1.000	1.021 (−0.345 to 2.342)	.123
Week 12	2.227 (0.474)	−0.267 (0.487)	<.001*/1.000	2.494 (1.091 to 3.741)	<.001*
Week 18	1.381 (0.478)	−0.761 (0.492)	.115/1.000	2.142 (0.768 to 3.505)	.002*
**SMI**					
Week 0					
Week 6	−0.020 (0.053)	−0.118 (0.052)	1.000/.721	0.097 (−0.049 to 0.244)	.194
Week 12	0.077 (0.053)	0.029 (0.055)	1.000/1.000	0.048 (−0.104 to 0.199)	.538
Week 18	0.198 (0.054)	0.001 (0.056)	.009*/1.000	0.200 (0.046 to 0.353)	.011*
**SPPB**					
Week 0					
Week 6	1.707 (0.268)	−0.692 (0.265)	<.001/.269	2.399 (1.661 to 3.138)	<.001*
Week 12	1.139 (0.271)	−1.329 (0.278)	.001*/<.001*	2.468 (1.707 to 3.228)	<.001*
Week 18	1.147 (0.273)	−1.829 (0.281)	.001*/<.001*	2.976 (2.209 to 3.744)	<.001*
**SarQoL**					
Week 0					
Week 6	5.420 (0.535)	−1.836 (0.528)	<.001*/.017	7.256 (5.782 to 8.730)	<.001*
Week 12	2.766 (0.541)	−2.809 (0.556)	<.001*/<.001*	5.574 (4.055 to 7.094)	<.001*
Week 18	0.435 (0.546)	−4.411 (0.574)	1.000/<.001*	4.846 (3.294 to 6.399)	<.001*
**CAF**					
Week 0					
Week 6	47.461 (10.1)	56.024 (10.3)	<.001*/<.001*	−8.56 (−36.9 to 19.80)	.555
Week 12					
Week 18	−5.860 (10.3)	55.911 (11.2)	1.000/<.001*	−61.77 (−91.5 to −32.01)	<.001*

Abbreviations: CAF, C-terminal agrin fragment; CI, confidence interval; CG, control group; HGS, hand grip strength; SarQoL, sarcopenia quality of life; *SE*, standard error; SMI, skeletal muscle index; SPPB, Short Physical Performance Battery.

* Significant difference.

#### SMI

At baseline, both groups were comparable (*p* = .848) with a mean score of 5.09 ± 1.15 vs 5.14 ± 1.01 in the IG and CG, respectively. The group*time interaction (*F* = 2.36, *p* = .072, η^2^_p_ = .033) was found to be not significant, with a small effect size. At 6 and 12 weeks, the change in SMI from baseline was not significant for the IG compared to the CG, with an estimate of 0.097 (95% CI, −0.05 to 0.24, *p* = .194) and 0.048 (95% CI, −0.10 to 0.19, *p* = .538), respectively. However, at 18 weeks, the IG had significantly greater SMI compared to the CG (estimate = 0.200; 95% CI, 0.05 to 0.35, *p* = .011). The ICC for individuals was 0.944, indicating most SMI variance was between persons. Cluster-level ICC was 0.875, showing moderate between-cluster variability. Residual variance (0.05) suggests the model explained SMI variability well. After adjusting for covariates, the group-by-time interaction at 18 weeks remained significant (estimate = 0.200, *p* = .011), confirming meaningful gains in SMI from the intervention. The interaction analysis using repeated measures ANOVA demonstrated that the three-way interaction (Time × Group × Sarcopenia status; *p* = .622) reached statistical significance. These findings indicate that changes in SMI over time were not differentially influenced by the intervention or sarcopenia status, suggesting that the stratified intervention did not introduce meaningful heterogeneity in treatment effects for this outcome. Post hoc comparisons are detailed in Table 3.

#### SPPB

At baseline, both the groups were not comparable (*p* = .047), with better physical performance in IG compared to CG (8.33 ± 2.72 vs 7.08 ± 2.77). There was significant group*time interaction (*F* = 24.07, *p* < .001, η^2^_p_ = .254) with a large effect size. At six weeks, the change in SPPB from baseline was significant for the IG compared to the CG, with an estimate of 2.40 (95% CI, 1.70 to 3.14, *p* < .001). Similar improvements were observed at 12 weeks (estimate = 2.47; 95% CI, 1.70 to 3.23, *p* < .001) and 18 weeks (estimate = 2.98; 95% CI, 2.21 to 3.74, *p* < .001). The ICC for individuals was 0.804, showing that most SPPB variance was between persons. Cluster ICC was 0.000, indicating no SPPB variability from clusters. Low residual variance (1.37) suggests the model explained SPPB well. After adjusting for covariates, the intervention effects on SPPB remained robust and significant at all time points (6, 12, and 18 weeks; all *p* < .001). Age showed a significant negative association with SPPB scores (*p* = .021), indicating reduced performance with increasing age. However, this did not alter the intervention’s effect, as group-by-time estimates were similar to the unadjusted model. For SPPB, the interaction analysis using the repeated measures ANOVA demonstrated that the three-way interaction (Time × Group × Sarcopenia status) was not significant (*p* = .764). This indicates that, despite differences in baseline severity and temporal patterns, the effectiveness of the intervention remained consistent across sarcopenia strata, thereby mitigating concerns regarding treatment heterogeneity. Post hoc comparisons are detailed in Table 3.

#### SarQoL

Baseline mean SarQoL scores (IG = 46.3 ± 14.3; CG = 49.7 ± 13.5) were comparable between both groups (*p* = .292). There was significant interaction between time and group (*F* = 34.038, *p* < .001, η^2^_p_ = .328) with a large effect size. The change in SarQoL score was significant for the IG compared to the CG at 6, 12, and 18 weeks. The greatest change from baseline between IG and CG, with an estimate of 7.25 (95% CI, 5.80 to 8.73, *p* < .001), was found at 6 weeks. Furthermore, the improvement remained significant at 12 weeks (estimate = 5.57; 95% CI, 4.05 to 7.10, *p* < .001), and 18 weeks (estimate = 4.84; 95% CI, 3.30 to 6.40, *p* < .001). The ICC for individuals was 0.966, showing that most SarQoL variance was between persons. Cluster ICC was 0.824, indicating moderate between-cluster variability. Residual variance (5.44) suggests the model explained SarQoL. After covariate adjustment, group-by-time interactions remained significant at all time points (all *p* < .001), confirming the intervention’s strong and sustained effect on SarQoL. Only daily physical activity was significantly associated with higher SarQoL scores (*p* = .007), but did not alter the intervention’s impact. For SarQoL, an interaction analysis using the repeated measures ANOVA demonstrated that the three-way interaction (Time × Group × Sarcopenia status) was not statistically significant (*p* = .537), suggesting that the intervention effect was consistent across sarcopenia categories. These findings, in conjunction with similar patterns observed for other key outcomes, indicate that stratification of the intervention did not introduce meaningful heterogeneity in treatment effects. Post hoc results are in Table 3.

#### CAF

Baseline mean CAF levels (IG = 79.2 ± 13.9; CG =77.0 ± 33.3) were comparable between both groups (*p* = .704). There was a significant group*time interaction (*F* = 9.36, *p* < .001, η^2^_p_ = .113) effect, with a moderate effect size. The interaction (group*time) effect indicates the difference in CAF change between groups at 6 weeks is not significant (estimate = −8.56; 95% CI, −36.9 to 19.80, *p* = .555). It suggests that at week 6, the increase in CAF was similar in both groups. Further, the interaction was found significant at week 18 (estimate = −61.77; 95% CI, −91.5 to −32.01, *p* < .001), meaning that at week 18, CAF levels in the IG had dropped much more compared to the CG. Individual variance was 281 (*SD* = 16.8, ICC = 0.1244), showing baseline CAF differences. Cluster variance was 214 (*SD* = 14.6, ICC = 0.0976), explaining 9.76% of CAF variance. Residual variance (1978, *SD* = 44.5) indicates substantial unexplained variability. After adjusting for covariates, the group-by-time interaction for CAF at 18 weeks remained significant (Estimate = −60.91, *p* < .001), confirming the intervention’s effect. No significant interaction was seen at 6 weeks (*p* > .5). Age was the only covariate associated with higher CAF levels (*p* = .008), but it did not influence the intervention outcome. The sustained CAF reduction reflects a true physiological response. For CAF, the interaction analysis using repeated measures ANOVA demonstrated that the three-way interaction (Time × Group × Sarcopenia status; *p* = .904) was not statistically significant. These findings indicate that CAF changes over time were not differentially influenced by the intervention or sarcopenia status, further supporting that the stratified intervention did not introduce meaningful heterogeneity in treatment effects. Post hoc details are in Table 3.

During the intervention period, one participant reported experiencing pain in the knee joint. However, no other participants reported any adverse symptoms or complaints. One of the participants in the CG expressed unwillingness to continue with the study, citing the lack of intervention as the reason for discontinuation, and was therefore allowed to withdraw, respecting participant autonomy. Additionally, the final follow-up assessments for two participants could not be conducted due to health-related concerns, as both presented with fever at the time. Given the primary focus on participant safety and well-being, their exclusion from the final assessment was deemed necessary to prevent any potential risks associated with further evaluation.

## Discussion

This cluster randomized trial was conducted among sarcopenic older adults residing in LTCS to examine the effect of the newly developed ReStart-S program on grip strength, muscle mass, physical performance, quality of life, and blood-based biomarker levels. The findings demonstrate that participants in the IG who underwent the 6-week ReStart-S program showed significant improvements across multiple outcomes compared to the CG, who continued their usual daily activities.

HGS, a widely recognized biomarker of aging and predictor of adverse health outcomes in older adults, showed significant improvement in the IG at 12 and 18 weeks, despite no significant difference at 6 weeks. This delayed response suggests that the physiological adaptations initiated during the 6-week ReStart-S intervention were amplified by continued exercise engagement during the follow-up period. This aligns with studies showing that resistance training typically requires 8-12 weeks to produce measurable strength gains in older adults.^33^ Early strength gains in older adults are typically attributed to neural responses, including enhanced motor unit recruitment, increased firing rates, and reduced antagonist coactivation.^34^^,^^35^ These responses improve the efficiency of force production without substantial changes in muscle morphology. However, sustained improvements such as those observed at 12 weeks are more likely driven by muscular adaptations, including hypertrophy and remodeling of muscle fibers, which require longer durations of mechanical loading to manifest.^36^ Continued exercise likely promoted the secretion of myokines such as Interleukin-6 (IL-6), Insulin Growth Factor-1 (IGF-1), and irisin, which play key roles in muscle regeneration, satellite cell activation, and neuromuscular junction integrity.^37^ These autocrine and paracrine signals enhance muscle protein synthesis and counteract sarcopenic processes, particularly in aging muscle where anabolic resistance is prevalent.^38^ Moreover, the behavioral reinforcement of exercise during the follow-up period may have contributed to habit formation and improved adherence. Older adults who perceive functional gains and experience positive affect during exercise are more likely to maintain activity levels, which in turn supports sustained physiological benefits.^39^ This behavioral continuity is critical, as long-term adherence is often the limiting factor in translating short-term interventions into meaningful health outcomes.

Although the group*time interaction for SMI was not statistically significant across all time points, a notable improvement in SMI at 18 weeks was observed in the IG compared to the CG. This delayed response aligns with the temporal dynamics of muscle hypertrophy, where early responses to exercise are predominantly neural, and measurable increases in muscle mass typically emerge after sustained training.^40^^,^^41^ The continuation of exercise by IG participants beyond the initial 6-week ReStart-S protocol likely contributed to the significant gains observed at 18 weeks. This supports evidence that longer-duration resistance or multicomponent training is necessary to overcome anabolic resistance and stimulate satellite cell activation, myofibrillar protein synthesis, and muscle fiber hypertrophy in older adults.^42^ The absence of significant changes at 6 and 12 weeks may reflect the lag in architectural remodeling, which requires consistent mechanical loading and metabolic stimulus over time.^43^ Meta-analyses have shown that while strength gains often precede changes in muscle mass, structural adaptations such as increased SMI are achievable with prolonged intervention.^44^ Some studies report functional improvements without corresponding muscle mass changes, underscoring the role of neuromuscular efficiency in early-phase adaptations.^45^ Our findings reinforce this trajectory, with SMI gains emerging only after extended engagement in the exercise regimen. It is also important to consider the measurement modality. SMI was derived using BIA, which directly does not measure the SMM rather estimates the percentage of skeletal muscle ratio, using which SMM and further SMI has been calculated. Although BIA is practical and noninvasive, it may lack the sensitivity to detect subtle changes in lean mass compared to dual-energy X-ray absorptiometry (DXA) or magnetic resonance imaging (MRI).^46^ This limitation could have attenuated the detection of early or modest changes in SMI, particularly at 6 and 12 weeks.

The significant group*time interaction observed for SPPB underscores the efficacy of the multicomponent exercise intervention in enhancing physical performance among sarcopenic older adults. Improvements in SPPB scores were evident as early as 6 weeks and continued to increase through 12 and 18 weeks, suggesting both rapid and sustained functional gains. These findings are consistent with prior research demonstrating that structured, progressive exercise programs can elicit meaningful improvements in mobility, balance, and lower limb strength in LTCS.^47^^,^^48^ Importantly, the intervention’s effect remained robust after adjusting for covariates, including age, which was negatively associated with SPPB scores. This suggests that while aging contributes to functional decline, tailored exercise interventions can mitigate its impact and promote resilience in physical performance. The magnitude of change in SPPB scores, ranging from +2.40 at 6 weeks to +2.98 at 18 weeks, exceeds the minimal clinically important difference (MCID) of 0.5-1.0 points reported in older populations^49^ reinforcing the clinical relevance of these gains. Notably, a substantial decline in SPPB scores was observed in the control group over the 18-week period. While the magnitude of this reduction may appear greater than typically reported, a consistent deterioration was evident across all SPPB domains, including balance, gait speed, and sit-to-stand performance, supporting the likelihood of a true functional decline rather than an isolated or spurious finding. This pattern was further corroborated by a parallel decline in HGS, indicating concurrent reductions in overall muscle function. Such changes may reflect the natural progression of sarcopenia and physical deconditioning in older adults receiving usual care in long-term care settings. This pattern may represent the natural history of untreated sarcopenia in LTCS residents, characterized by progressive deterioration in physical function over time. Therefore, the benefits of the ReStart-S intervention may extend beyond functional improvement alone, potentially helping to arrest or slow the rapid decline typically observed in older adults receiving usual care. Nevertheless, the extent of decline should be interpreted with caution, given variability in reported trajectories across similar populations.

Sarcopenia is increasingly recognized not only for its physical consequences but also for its profound impact on HRQoL.^31^ In this study, the use of the SarQoL-Kannada version enabled culturally and linguistically appropriate assessment of QoL in long-term care residents, revealing significant improvements in the IG across all time points compared to controls.^32^ The early and sustained improvements in SarQoL scores, most pronounced at 6 weeks and persisting through 12 and 18 weeks, suggest that the intervention had a meaningful and lasting effect on participants’ perceived well-being. Physiologically, these improvements may be attributed to enhanced muscle strength, improved mobility, and reduced fatigue, all of which are known to influence domains within the SarQoL domains.^50^ The observed change exceeded the smallest detectable change threshold, confirming a true effect.^51^ Exercise interventions, particularly those incorporating resistance and functional training, promote neuromuscular adaptations, increase mitochondrial efficiency, and reduce systemic inflammation, all of which contribute to improved physical function and psychosocial well-being. Additionally, the group-based nature of the intervention within the institutional setting may have provided psychosocial benefits through increased social interaction, peer support, motivation, and behavioral reinforcement, which could have further contributed to the observed improvements in SarQoL scores beyond the biological effects of exercise alone.^52^ To our knowledge, this is the first intervention trial to assess changes in SarQoL scores following exercise in this population.

Given the influence of motivation on functional outcomes, CAF, a blood-based biomarker, offers valuable insights into intervention effects. This study is the first to explore exercise effects on CAF in sarcopenic LTCS residents. While both groups showed initial CAF increases at 6 weeks, this early rise likely reflects an acute mechanical stress response or proteolytic transient response, which could be in response to transient neuromuscular junction (NMJ) destabilization due to exercise-induced stress or ongoing sarcopenic degeneration, consistent with prior findings that acute physical activity can elevate CAF levels through increased agrin cleavage by neurotrypsin.^53^ However, the biologically relevant signal was the significant reduction in CAF levels observed at 18 weeks in the intervention group compared to controls, suggesting a sustained restoration of NMJ stability. This decline likely reflects reduced agrin fragmentation, improved anchoring of acetylcholine receptors, and enhanced motor neuron-muscle fiber connectivity, hallmarks of NMJ remodeling following structured resistance exercise.^30^^,^^54^ This pattern suggests early NMJ remodeling followed by stabilization, echoing findings from other exercise studies.^55^^,^^56^ Such adaptations are critical in aging populations, where NMJ degradation contributes to muscle atrophy and functional decline.^54^ Overall, the delayed but significant reduction in CAF levels in the intervention group underscores CAF’s utility as a dynamic biomarker for NMJ remodeling. These findings support its role in monitoring sarcopenia interventions, particularly those targeting neuromuscular integrity through progressive resistance training.

The ReStart-S program is a novel, multicomponent exercise intervention co-designed specifically for sarcopenic older adults in LTCS.^18^ Unlike existing guidelines focused on community-dwelling older adults and lacking co-designing,^9–11^ A co-designed ReStart-S addresses the unique needs of institutionalized populations. Its structured progression based on sarcopenia severity allows for individualized adaptation, making it both targeted and practical for this setting. However, India’s LTCS faces significant challenges compared to the Western care model in implementing and adhering to reablement due to cultural, economic, and resource-related factors. In addition, sociocultural factors in India, including strong family-centered caregiving traditions and limited acceptance of institutional care, may influence participant engagement, adherence, and reablement outcomes.^20^ Resource constraints and variability in institutional support may further affect the implementation and sustainability of structured exercise interventions. Therefore, the present findings provide important context-specific evidence supporting the feasibility and effectiveness of ReStart-S among sarcopenic older adults residing in Indian LTCS settings.

Strengths and limitations: The major strength of the present study is to examine the effect of ReStart-S, on CAF levels among sarcopenic older adults residing in LTCS. Furthermore, the study looked at the change in QoL using the SarQoL questionnaire, which has not been used in interventional trials. In addition to that the ReStart-S program, which has been designed specifically for sarcopenic older adults residing in LTCS, has been tested for its effectiveness on various outcome measures. However, the research has some limitations that warrant attention. First, participants nutritional status was not directly assessed; instead, weight and body composition were measured using BIA as proxy indicators. Second, SMI was also estimated using BIA, which, despite being convenient and feasible in large-scale or clinical settings, lacks the sensitivity to detect small changes in muscle mass and has lower reliability compared to gold-standard imaging techniques like DXA or MRI. Third, despite statistical adjustment, the baseline differences in age, education, and activity levels between groups may have introduced residual confounding, potentially biasing the estimated intervention effects. Fourth, since adherence during follow-up was self-reported, potential recall and social desirability biases may have led to overestimation of exercise engagement, thereby influencing the interpretation of sustained effects at 12 and 18 weeks. Additionally, loss to follow-up due to health concerns or unwillingness to continue may have introduced selective survival bias, potentially resulting in 18-week outcomes that reflect a relatively healthier subset of participants. Fifth, the uneven distribution of clusters between the intervention and control groups may have introduced bias and limited generalizability. To address this, we re-grouped clusters to balance participant numbers and applied linear mixed models and intraclass correlation analysis to account for variability and enhance the robustness of our findings. Finally, a post-hoc power analysis based on the actual data indicated that the study had about 68% power to detect the observed effect, implying a 32% chance of Type II error. Therefore, the findings should be interpreted with some caution.

Future recommendations: Future trials should consider adding a structured nutritional intervention to complement the ReStart-S program for greater effectiveness in managing sarcopenia. Extending the program to 12 weeks by running each weekly phase over 14 days may support better physiological adaptation and long-term outcomes. Additionally, using stratified randomization with weighted distribution can help minimize cluster imbalances.

## Conclusion

The ReStart-S, a multi-component exercise program designed specifically for sarcopenic older adults residing in LTCS, has been found to be effective. There was found to be an improvement in grip strength, physical performance, QoL, and a reduction in biomarker levels. Notably, the program exhibited a delayed effect, with most improvements observed at the 12-week mark, hence highlighting the need for longer-duration intervention programs for sarcopenic older adults.

## Supplementary material

Supplementary material is available at *The Journals of Gerontology, Series A: Biological Sciences and Medical Sciences* online.

## Supplementary Material

glag141_Supplementary_Data

## Data Availability

The datasets used and/or analyzed during the current study are available from the corresponding author upon reasonable request.
