# Supplementary material for: Cluster randomized trial of reablement strategies targeting sarcopenia (ReStart-S) in long-term care settings
Source: J Gerontol A Biol Sci Med Sci. 2026 May 29;81(7):glag141. doi: 10.1093/gerona/glag141 (PMC13287990; doi:10.1093/gerona/glag141)
Supplement: glag141_Supplementary_Data [file glag141_supplementary_data.zip › 13-Jun-2026_044556_Supplementary_material_1.docx]

**Title:** Cluster Randomized Trial of Reablement Strategies Targeting Sarcopenia (ReStart-S) in Long-Term Care Settings

**Supplementary material 1**

**Consort 2010 checklist of information to include when reporting a cluster randomized trial**

| Item | Standard checklist item | Extension for cluster design | Page number |
| --- | --- | --- | --- |
| Title and abstract | | | |
| 1a | Identification as a randomised trial in the title | Identification as a cluster randomised trial in the title | 1 |
| 1b | Structured summary of trial design, methods, results, and conclusions See table 2  (for specific guidance see CONSORT for abstracts) |  | 4 |
| Introduction | | | |
| Background and objectives | | | |
| 2a | Scientific background and explanation of rationale | Rationale for using a cluster design | Page number 6 |
| 2b | Specific objectives or hypotheses | Whether objectives pertain to the cluster level, the  individual participant level, or both | Page number 6-7 |
| Methods | | | |
| Trial design | | | |
| 3a | Description of trial design (such as parallel, factorial) including  allocation ratio | Definition of cluster and description of how the design features apply to the clusters | Page number 8 |
| 3b | Important changes to methods after trial commencement (such as  eligibility criteria), with reasons |  | Not applicable |
| Participants | | | |
| 4a | Eligibility criteria for participants | Eligibility criteria for clusters | Page number 8-9 |
| 4b | Settings and locations where the data were collected |  | Page number 8 |
| Interventions | | | |
| 5 | The interventions for each group with sufficient details to allow  replication, including how and when they were actually administered | Whether interventions pertain to the cluster level, the  individual participant level, or both | Page number 10-11 |
| Outcomes | | | |
| 6a | Completely defined prespecified primary and secondary outcome  measures, including how and when they were assessed | Whether outcome measures pertain to the cluster  level, the individual participant level, or both | Page number 13-15 |
| 6b | Any changes to trial outcomes after the trial commenced, with  Reasons |  | Not applicable |
| Sample size | | | |
| 7a | How sample size was determined | Method of calculation, number of clusters(s) (and  whether equal or unequal cluster sizes are assumed),  cluster size, a coefficient of intracluster correlation  (ICC or k), and an indication of its uncertainty | Page number 9 |
| 7b | When applicable, explanation of any interim analyses and stopping  Guidelines |  | Not applicable |
| Randomization | | | |
| Sequence generation | | | |
| 8a | Method used to generate the random allocation sequence |  | Not applicable |
| 8b | Type of randomisation; details of any restriction (such as blocking and block size) | Details of stratification or matching if used | Page number 10 |
| Allocation concealment mechanism | | | |
| 9 | Mechanism used to implement the random allocation sequence  (such as sequentially numbered containers), describing any steps  taken to conceal the sequence until interventions were assigned | Specification that allocation was based on clusters  rather than individuals and whether allocation  concealment (if any) was at the cluster level, the  individual participant level, or both | Not applicable |
| Implementation | | | |
| 10 | Who generated the random allocation sequence, who enrolled participants, and who assigned participants to interventions | Replaced by 10a, 10b, and 10c | Not applicable |
| 10a |  | Who generated the random allocation sequence, who  enrolled clusters, and who assigned clusters to  interventions | Not applicable |
| 10b |  | Mechanism by which individual participants were  included in clusters for the purposes of the trial (such  as complete enumeration, random sampling) | Not applicable |
| 10c |  | From whom consent was sought (representatives of  the cluster, or individual cluster members, or both)  and whether consent was sought before or after  randomisation | Page number 9-10 |
| Blinding | | | |
| 11a | If done, who was blinded after assignment to interventions (for  example, participants, care providers, those assessing outcomes)  and how |  | Page number 12 |
| 11b | If relevant, description of the similarity of interventions |  | Not applicable |
| Statistical methods | | | |
| 12a | Statistical methods used to compare groups for primary and secondary outcomes | How clustering was taken into account | Page number 16 |
| 12b | Methods for additional analyses, such as subgroup analyses and  adjusted analyses |  | Not applicable |
| Results | | | |
| Participant flow (a diagram is  strongly recommended): | | | |
| 13a | For each group, the numbers of participants who were randomly  assigned, received intended treatment, and were analysed for the  primary outcome | For each group, the numbers of clusters that were  randomly assigned, received intended treatment, and  were analysed for the primary outcome | Page number 17 |
| 13b | For each group, losses and exclusions after randomisation, together  with reasons | For each group, losses and exclusions for both  clusters and individual cluster members | Page number 17 |
| Recruitment | | | |
| 14a | Dates defining the periods of recruitment and follow-up |  | Page number 13 |
| 14b | Why the trial ended or was stopped |  |  |
| Baseline data | | | |
| 15 | A table showing baseline demographic and clinical characteristics  for each group | Baseline characteristics for the individual and cluster  levels as applicable for each group | Page number 18 and 39 |
| Numbers analysed | | | |
| 16 | For each group, number of participants (denominator) included in  each analysis and whether the analysis was by original assigned  groups | For each group, number of clusters included in each  Analysis | Page number 18-22 |
| Outcomes and estimation | | | |
| 17a | For each primary and secondary outcome, results for each group,  and the estimated effect size and its precision (such as 95%  confidence interval) | Results at the individual or cluster level as applicable  and a coefficient of intracluster correlation (ICC or k)  for each primary outcome | Page number 18-22 |
| 17b | For binary outcomes, presentation of both absolute and relative  effect sizes is recommended |  | Not applicable |
| Ancillary analyses | | | |
| 18 | Results of any other analyses performed, including subgroup  analyses and adjusted analyses, distinguishing prespecified from  exploratory |  | Not applicable |
| Harms | | | |
| 19 | All important harms or unintended effects in each group (for specific  guidance see CONSORT for harms106) |  | Page number 22 |
| Discussion | | | |
| Limitations | | | |
| 20 | Trial limitations, addressing sources of potential bias, imprecision,  and, if relevant, multiplicity of analyses |  | Page number 28-29 |
| Generalisability | | | |
| 21 | Generalisability (external validity, applicability) of the trial findings | Generalisability to clusters and/or individual  participants (as relevant) | Page number 29 |
| Interpretation | | | |
| 22 | Interpretation consistent with results, balancing benefits and harms,  and considering other relevant evidence |  | Page number 23-28 |
| Other Information | | | |
| Registration | | | |
| 23 | Registration number and name of trial registry |  | Page number 8 and 30 |
| Protocol | | | |
| 24 | Where the full trial protocol can be accessed, if available |  | Page number 8 |
| Funding | | | |
| 25 | Sources of funding and other support (such as supply of drugs), role  of funders |  | - |
